# Highlight report: The EDI3-GPAM axis in tumor cell migration

**DOI:** 10.17179/excli2017-855

**Published:** 2017-10-19

**Authors:** Reham Hassan

**Affiliations:** 1 Forensic Medicine and Toxicology Department, Faculty of Veterinary Medicine, South Valley University, Qena, Egypt

## ⁯

Recently, the glycerophosphodiesterase EDI3 (GPCPD1) has been shown to represent a key factor of choline metabolism that mediates tumor cell migration, adhesion and spreading (Stewart et al., 2012[16]; Lesjak et al., 2014[9]). EDI3 cleaves glycerophosphocholine to choline and glycerol-3-phosphate (Marchan et al., 2012[10]). Choline is further metabolized by choline kinase alpha (CHKA) that generates phosphocholine. The second EDI3 product, glycerol-3-phosphate (G3P) is a substrate of glycerol-3-phosphate acyltransferase 1 (GPAM) that generates phosphatidic acid (PA). Currently, it remains unclear, whether the pathway via CHKA or GPAM (or both) is relevant for increased tumor cell migration. 

Recently, the group of Rosemarie Marchan at Dortmund University has clarified this question (Marchan et al., 2017[10]). The authors overexpressed and knocked down GPAM and CHKA in several ovarian cancer cell lines. Interestingly, they demonstrated that only manipulation of GPAM and not CHKA influenced cell migration. Moreover, silencing GPAM reduced the growth of mouse tumor xenografts (Marchan et al., 2017[10]). High GPAM expression was also associated with worse prognosis in ovarian cancer patients. Therefore, the study of Marchan and colleagues clearly demonstrated that GPAM is the relevant enzyme downstream of EDI3 responsible for enhanced tumor cell migration. Altered metabolism of tumor cells has been linked to progression and worse outcome in numerous studies (Currie et al., 2013[2]; Pavlova and Thompson, 2016[12]; Santos and Schulze, 2012[13]). However, choline metabolism has attracted comparatively little attention (Glunde et al., 2015[3]; Okazaki et al., 2010[11]; Granata et al., 2014[4]; Hu et al., 2016[8]). Therapy and subtyping of carcinomas depends on the identification of factors influencing tumor prognosis (Heimes et al., 2017[5][6]; Hellwig et al., 2016[7]; Stock et al., 2015[17]; Cadenas et al., 2014[1]; Sicking et al., 2014[15]; Shakeri et al., 2016[14]). The present study of Marchan and colleagues (2017[10]) demonstrates that the EDI3-GPAM pathway in choline metabolism influences the tumor phenotype and prognosis, therefore justifying further research how exactly these enzymes link choline metabolism to tumor cell migration. 
